# Data for the ins and outs of involuntary part-time employment

**DOI:** 10.1016/j.dib.2020.106686

**Published:** 2020-12-26

**Authors:** Daniel Borowczyk-Martins, Etienne Lalé

**Affiliations:** a Department of Economics, Copenhagen Business School, Porcelaenshaven 16A, 2000 Frederiksberg, Denmark; b Department of Economics, Université du Québec à Montréal, C.P. 8888, Succ. centre ville, Montréal (QC) H3C 3P8, Canada

**Keywords:** Involuntary part-time employment, Unemployment, Labor market flows, Business cycles

## Abstract

Data are US monthly time series of involuntary part-time employment stocks and flows from 1976 until 2019 (covering five economic downturns), derived from the US Current Population Survey (CPS). Stocks and flows are cleared from discrepancies over time caused by the 1994 redesign of the CPS, and they are adjusted to control for margin error problems and time aggregation biases. Data are available in two different formats: unadjusted and adjusted for misclassification errors – another important sources of biases in worker flows data. The time series obtained through these adjustments allow for a comprehensive account of the cyclical dynamics of involuntary part-time employment.

## Specifications Table

**Table:** 

Subject	Macroeconomics
Specific subject area	Macroeconomics and labor economics: Measurement of labor market dynamics through worker flows data
Type of data	Table
	Graph
How data were acquired	The raw data are microdata from the U.S. Current Population Survey. These data are publicly and freely available from the website of the U.S. Census Bureau (https://www.census.gov/programs-surveys/cps.html).
Data format	Raw data can be downloaded from the website of the U.S. Bureau of Census (https://www.census.gov/programs-surveys/cps.html). We use the raw data to construct time series of involuntary part-time employment stocks and flows from 1976 until 2019 cleared from several statistical biases.
Parameters for data collection	The data collection process ensures that data are representative of the working-age population of the United States.
Description of data collection	Data are collected through a household labour force survey.
Data source location	Website of the U.S. Census Bureau (https://www.census.gov/programs-surveys/cps.html).
Data accessibility	Data are available in the Mendeley repository: https://data.mendeley.com/datasets/czf7f53xt5/1.
Related research article	D. Borowczyk-Martins, E. Lalé, The ins and outs of involuntary part-time employment, Labour Economics, Vol. 67, 101940, December 2020. https://doi.org/10.1016/j.labeco.2020.101940[1].

## Value of the Data

• Data are transition probabilities of moving across full-time employment, voluntary part-time employment, involuntary part-time employment, unemployment, and nonparticipation. Data are calculated at the monthly frequency and cover U.S. workers over the period from 1976 until 2019. These data are useful to assess the role of involuntary part-time employment in shaping the dynamics of the U.S. labour market.
• Worker flows across labour market states are key source of empirical evidence to understand aggregate labour market dynamics. Previous research has shown that part-time employment, and more specifically involuntary part-time work, plays a major role in fluctuations in hours per worker. Thus, these data are relevant for a wide range of researches in macroeconomics and labour economics.
• Data can be used to calculate moments (averages, standard deviation, correlation, etc.) of different transition probabilities over a long period of time and at a relatively high frequency. These moments can be used to inform the calibration of models of cyclical labour adjustments. They can also be used to assess the validity of these models, by comparing whether they reproduce the cyclical behaviour of transition probabilities.

## 1 Data Description

Data are transition probabilities of moving across full-time employment, voluntary part-time employment, involuntary part-time employment, unemployment, and nonparticipation. Data are calculated at the monthly frequency and cover U.S. workers over the period from 1976 until 2019. The content of each *_baseline MS Excel data file is as follows: time series of seasonally adjusted stocks (normalized by the corresponding population size), and time-series of seasonally adjusted transition probabilities, corrected for margin error and time aggregation bias. The content of each *_reclassified MS Excel data file is identical, but transition probabilities are in addition adjusted for potentially spurious transitions. The seasonal adjustment is made using the U.S. Census Bureau's X-13ARIMA-SEATS program. The correction for margin error is the standard procedure called “raking”, which reconciles the changes in stocks predicted by the transition probabilities (calculated using longitudinally linked data) with the actual changes in stocks (calculated using cross-sectional data). The adjustment for time aggregation bias is also standard and is based on Shimer [2]’s continuous-time correction.

## 2 Experimental Design, Materials and Methods

The raw CPS data are downloaded from the website of the U.S. Bureau of Census (https://www.census.gov/programs-surveys/cps.html). To import the raw data into Stata, we use the dictionary files provided on the National Bureau of Economic Research (NBER) webpage: http://www2.nber.org/data/cps_basic_progs.html. To construct worker flows data, individual records must be longitudinally linked, which can be done using the Stata programs provided on this NBER webpage: http://www2.nber.org/data/cps_match.html.

The statistical corrections applied to the data are as follows

• We correct pre-1994 data to control for the redesign of the CPS that took place in January 1994:
  ○ We adjust the levels of the series of monthly stocks by combining them with the series of part-time employment stocks calculated using the Annual Social and Economic Supplements (ASEC) of the CPS. What this correction does is ensure that the close co-movement between the post-1994 ASEC and monthly time series is the same in the pre-1994 period.
  ○ We correct the pre-1994 flows by targeting the dynamics of the series of stocks estimated in the previous step using a margin-error (or raking) procedure. This adjustment reconciles the predicted changes in stocks with the actual changes in stocks that occur between two consecutive months.
• We correct transition probabilities to control for time aggregation bias. Time-aggregation bias refers to the discrepancy between the transition probabilities measured at discrete intervals and the underlying continuous process which they seek to measure. Specifically, the competing risks structure of the process implies that the discrete-time (monthly) probabilities miss some of the transitions that occur at a higher frequency. We adapt Shimer [2]’s continuous-time correction to our setup to address this bias.

## Ethics Statement

N/A.

## Credit author statement

Daniel Borowczyk-Martins: design of methodology; implementation of the computer code; application of statistical techniques to analyse the data; preparation, creation and presentation of the published work.

Etienne Lalé: design of methodology; implementation of the computer code; application of statistical techniques to analyse the data; preparation, creation and presentation of the published work.

## Declaration of Competing Interest

The authors declare that they have no known competing financial interests or personal relationships which have or could be perceived to have influenced the work reported in this article.
